# Trust-Aware Causal Consistency Routing for Quantum Key Distribution Networks Against Malicious Nodes

**DOI:** 10.3390/e27111100

**Published:** 2025-10-24

**Authors:** Yi Luo, Qiong Li

**Affiliations:** School of Cyberspace Science, Faculty of Computing, Harbin Institute of Technology, Harbin 150001, China; yi_luo@stu.hit.edu.cn

**Keywords:** quantum key distribution networks, routing, malicious nodes

## Abstract

Quantum key distribution (QKD) networks promise information-theoretic security for multiple nodes by leveraging the fundamental laws of quantum mechanics. In practice, QKD networks require dedicated routing protocols to coordinate secure key distribution among distributed nodes. However, most existing routing protocols operate under the assumption that all relay nodes are honest and fully trustworthy, an assumption that may not hold in realistic scenarios. Malicious nodes may tamper with routing updates, causing inconsistent key-state views or divergent routing plans across the network. Such inconsistencies increase routing failure rates and lead to severe wastage of valuable secret keys. To address these challenges, we propose a distributed routing framework that combines two key components: (i) Causal Consistency Key-State Update, which prevents malicious nodes from propagating inconsistent key states and routing plans; and (ii) Trust-Aware Multi-path Flow Optimization, which incorporates trust metrics derived from discrepancies in reported states into the path-selection objective, penalizing suspicious links and filtering fabricated demands. Across 50-node topologies with up to 30% malicious relays and under all three attack modes, our protocol sustains a high demand completion ratio (DCR) (mean 0.90, range 0.81–0.98) while keeping key utilization low (16.6 keys per demand), decisively outperforming the baselines—Multi-Path Planned (DCR 0.48, 30.8 keys per demand) and OSPF (DCR ≤0.12, 296 keys per demand; max 1601). These results highlight that our framework balances reliability and efficiency, providing a practical and resilient foundation for secure QKD networking in adversarial environments.

## 1. Introduction

Quantum key distribution (QKD) is based on the principles of quantum uncertainty and quantum no-cloning, ensuring information-theoretic security (ITS) through physical laws [1]. To expand the application of QKD, numerous researchers have endeavored to construct QKD networks. A QKD network organizes a large number of QKD devices into a network, where they cooperate with each other to enable information-theoretic secure key distribution [2,3,4,5] over a wider area. At the current stage, demonstration experiments for QKD networks are becoming increasingly mature, with many successful experimental demonstrations based on trusted relay QKD networks [6,7,8,9,10].

An important challenge in QKD networks lies in the efficient allocation of limited key resources [11,12,13]. Since the current key generation rates of practical QKD systems remain far below the demands of large-scale applications [14,15,16,17], the problem of coordinating key distribution among multiple nodes becomes critical [14,15,18,19,20,21]. This issue is typically addressed through dedicated routing protocols [15,16,18,22,23], which determine both the end-to-end distribution paths and the amount of keys allocated along each path. Existing routing protocols, however, operate under the assumption that all relay nodes are fully trusted [15,16,18,21,22,23]. Under this assumption, each relay node is expected to execute the routing decisions faithfully and exactly as prescribed by the protocol.

However, this assumption is easily undermined in a practical QKD network [24,25]. This means we cannot ensure that these nodes will consistently operate as expected without eavesdropping or recording any secret information. [26] highlighted a weakness in QKD systems, where the associated security analyses assume that classical units are trusted. The study proposed a solution that ensures information-theoretic security even in the presence of cheaters. Furthermore, [27] examined scenarios involving a subset of malicious nodes, providing a security analysis for cases where multiple malicious nodes either collude or adopt active attack strategies. The authors of [28,29] approached the problem from a disjoint multiple paths perspective, presenting a key distribution scheme that accounts for scenarios where Eve can manipulate or compromise intermediate trusted nodes. In [15], a routing strategy was proposed to minimize the number of trusted relays involved in the distribution process. However, this approach still cannot tolerate the presence of untrusted relay nodes.

The studies mentioned above focus primarily on minimizing the impact of malicious nodes during the key distribution phase. However, current research on QKD networks has not yet explored routing protocols in the presence of malicious nodes. Therefore, these routing protocols are largely ineffective against security threats posed by malicious nodes. When these relay nodes are malicious, they may disregard routing table information entirely and act solely in their own self-interest. This behavior significantly reduces routing efficiency and substantially increases the probability of routing failures.

Typically, the security assumptions of classical network routing differ from those of QKD networks. Classical routing protocols were engineered under the assumption that all intermediate routers within an administrative domain are honest and physically secure [30,31]. They rely instead on end-to-end security mechanisms at higher layers to protect data integrity and confidentiality—for example, the Transport Layer Security (TLS) protocol at the application/transport layer and IPsec at the network layer [32,33]. Since standard asymmetric cryptosystems underlying those mechanisms are vulnerable to quantum attacks (e.g., Shor’s algorithm [34]), the classical security stack cannot be assumed as-is in quantum-threatened settings.

The key contribution of this work is not to minimize reliance on trusted relays, but to tolerate and contain the disruptive impact of malicious nodes on routing while keeping key utilization efficiency near its optimal level. Prior studies that reduce trust assumptions or employ multi-path routing still presuppose all honest relays that do not falsify routing information; even a small fraction of malicious relays can sharply degrade routing success under such assumptions. In contrast, we explicitly model adversarial relays that may misreport link state and mislead path selection, and we design a mechanism that preserves a consistent network view and robust path choices under partial adversarial presence. Absent extra advantages—such as synchronous timing guarantees, pre-shared end-to-end keys, or end-to-end entanglement resources—the control/routing plane of a QKD network is subject to classical Byzantine fault-tolerance bound [35] in distributed networks (the number of malicious nodes must be strictly below 1/3 of the total number of nodes). Our main contributions are summarized as follows:

1. We propose a Causal Consistency Key-State Update mechanism, enabling all nodes in the network to reach consensus regarding the key states of each link. Routing decisions are then made based on these results, effectively preventing honest nodes from misjudging the routing states of other links.
2. We develop a Trust-Aware Multi-path Flow Optimization framework. Within the Causal Consistency Key-State Update process, discrepancies between the link-state information reported by the two endpoints of the same link are computed. These discrepancies are used as quantitative indicators of the trustworthiness of the corresponding links and are incorporated into a new multi-path routing optimization model.
3. Extensive simulations on 50-node topologies with malicious-node ratios up to 30% demonstrate the significant superiority of our approach: the proposed protocol consistently sustains high demand completion ratios (DCR ≥0.90) while keeping key utilization efficiency (KUE) as low as ≈16.6 keys per demand. In contrast, Multi-Path Planned achieves only moderate performance (DCR ≈0.48, KUE ≈30.8), and OSPF collapses with extremely poor reliability (DCR ≈0.12) and prohibitive inefficiency (KUE up to 1601 keys per demand). These results highlight that our design achieves an order-of-magnitude improvement in efficiency and reliability across all attack modes.

The remainder of this paper is organized as follows. Section 2 introduces the classical optimization models for routing and outlines the security assumptions regarding malicious nodes. Section 3 presents our proposed Trust-Aware Causal Consistency Routing scheme designed to resist malicious nodes. Section 4 describes the simulation setup and discusses the experimental results. Finally, Section 5 concludes the paper.

## 2. Preliminary

### 2.1. Routing Problem in QKD Networks

We are considering a scenario where the QKD network is modeled as a graph G(V,E). The task of routing in a QKD network is to identify optimal paths between non-adjacent nodes, ensuring that a greater proportion of the QKD keys are utilized for key distribution rather than being consumed in the authentication of routing protocol messages.

Here, the routing requirement is denoted as $m_{ij}$, representing the amount of secret key *m* distributed from node *i* to node *j*. In general, a set of communication demands exists simultaneously, which can be represented as $R=\{m_{ij}|\left(i,j\right)\in P\}$, where P represents the set of node pairs requiring communication. Let $K_{e}$ denote the key consumption on link *e*. The allocation of $K_{e}$ is dependent on the key status of the link. The link state is typically represented by the key pool storage $S_{e}$. To prevent key exhaustion, a threshold value $S_{\min}$ is generally imposed on the key pool.

The original routing problem can be formulated as an optimization problem, where the objective is to decide the key consumption on each link to satisfy these communication demands while minimizing the total key consumption across all links. The optimization problem can be expressed as follows:

(1) $$\begin{array}{c} \min\sum_{t}\sum_{e\in E}K_{e}^{t} \end{array}$$

Here are the constraints:

1. Flow Conservation Constraint: For each node *v*, the total incoming and outgoing key flows must balance, except at the source and destination nodes:
  (2) $$\begin{array}{c} \sum_{e_{in}\in v_{in}}f_{e_{in}}^{t}-\sum_{e_{out}\in v_{out}}f_{e_{out}}^{t}=\sum_{(i,j)\in P,j=v}m_{ij}^{t}-\sum_{(i,j)\in P,i=v}m_{ji}^{t} \end{array}$$
  $f_{e_{in}}^{t}$ represents the incoming key flow on link *e* at time *t*.
  $f_{e_{out}}^{t}$ represents the outgoing key flow on link *e* at time *t*.
  $v_{in}$ denotes the set of incoming links to node *v*.
  $v_{out}$ denotes the set of outgoing links from node *v*.
  $m_{ij}^{t}$ represents the key demand from source *i* to destination *j* at time *t*.
2. Key Consumption on Links: The total key consumption on link *e* is given by
  (3) $$\begin{array}{c} K_{e}^{t}=f_{e_{in}}^{t}+f_{e_{out}}^{t} \end{array}$$
3. Key Pool Dynamics: The key pool storage $S_{e}$ generally varies dynamically over time, as it depends on both the generated and consumed key amounts at each time step. This can be expressed as
  (4) $$\begin{array}{c} S_{e}^{t+1}=S_{e}^{t}+G_{e}^{t}-K_{e}^{t} \end{array}$$
  $S_{e}^{t}$ is the key pool storage at time *t*.
  $G_{e}^{t}$ represents the key generation rate on link *e*.
  $K_{e}^{t}$ is the total key consumption on link *e*.
4. Key Storage Constraint: The key consumption should respect the available key storage while maintaining a minimum threshold:
  (5) $$\begin{array}{c} 0\leq K_{e}^{t}\leq S_{e}^{t}-S_{\min} \end{array}$$

To assess the effectiveness of different routing protocols in QKD networks, we define two fundamental performance metrics: demand completion ratio (DCR) and key utilization efficiency (KUE). We first define the QKD routing demand completion ratio (DCR) as the ratio of successfully completed communication demands to the total number of communication demands.

**Definition** **1** (Demand Completion Ratio)**.** *Let the demand completion ratio (DCR) at time t be defined as*

(6) $$DCR\left(t\right)=\frac{\sum_{(i,j)\in P}{\hat{m}}_{ij}^{t}}{\sum_{(i,j)\in P}m_{ij}^{t}},$$

*where $m_{ij}^{t}$ denotes the key demand from source i to destination j at time t, and  ${\hat{m}}_{ij}^{t}\leq m_{ij}^{t}$ denotes the portion of the demand that is successfully fulfilled using the available key resources and routing paths. P is the set of all source–destination pairs with active demands at time t.*

In addition, we define the key utilization efficiency (KUE) as the ratio of the total amount of key material consumed to the number of successfully completed communication demands. This metric reflects the average amount of key resources required to satisfy one communication demand, thereby serving as an indicator of the efficiency of key consumption in the network.

**Definition** **2** (Key Utilization Efficiency)**.** *Let the key utilization efficiency (KUE) at time t be defined as*

(7) $$KUE\left(t\right)=\frac{\sum_{(i,j)\in P}c_{ij}^{t}}{\sum_{(i,j)\in P}{\hat{m}}_{ij}^{t}},$$

*where $c_{ij}^{t}$ denotes the total amount of key material consumed to serve the demand from source i to destination j at time t, and ${\hat{m}}_{ij}^{t}$ denotes the portion of that demand successfully completed using available key resources and routing paths. P is the set of all source–destination pairs with active demands at time t.*

### 2.2. Malicious Nodes Assumption

Malicious Nodes Assumption: We assume that there are *f* malicious nodes in the QKD network. Unless honest nodes obtain concrete evidence of protocol violations, they cannot distinguish which nodes are malicious. Malicious nodes may collaborate and share the status of all QKD links connected to them, to intercept as much end-to-end key information as possible.

In our assumption, the malicious nodes have access to the key material associated with the malicious nodes, but the key material of the honest nodes can only be accessed by the attacker through actual attacks on the underlying quantum key distribution protocol. Since the probability of an attacker successfully compromising the underlying protocol of an honest node without relying on additional information is extremely low, we temporarily exclude scenarios where the attacker might obtain the honest node’s key material through optical or physical means. (Even if such an event occurs, we treat it as if the number of malicious nodes has increased by one.) For this work, we assume that the attacker has access only to the key material of all malicious nodes.

Malicious nodes can adopt a range of adversarial strategies to undermine the aforementioned optimization conditions, thereby degrading the success rate of key distribution or diminishing key utilization efficiency within the routing protocol.

**Figure 1:**
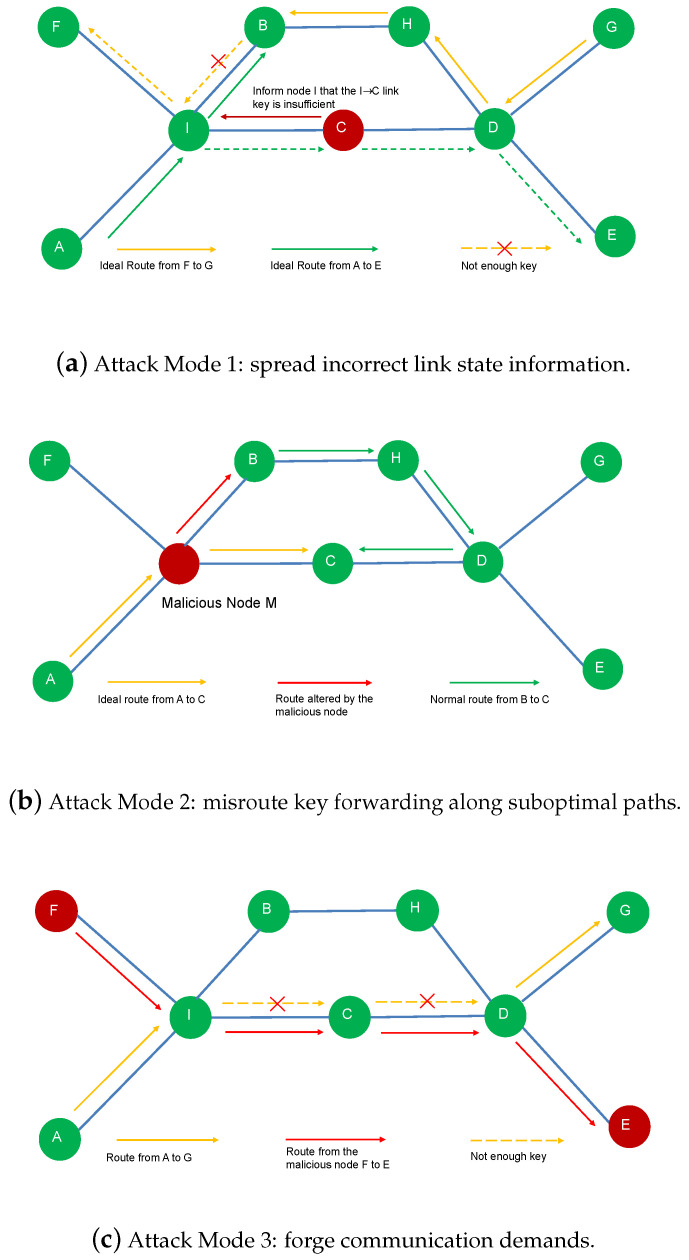
Three simple malicious node attack modes. The detail can be found in the paragraph “Detailed Explanation of Figure” above. Attack 1—malicious nodes spread incorrect link state information, leading to inconsistent global views. Attack 2—malicious nodes make routing decisions in their own way, misrouting key forwarding along suboptimal paths. Attack 3—malicious nodes forge communication demands, which lower the achievable DCR for honest nodes.

**Detailed Explanation of Figure 1:** Figure 1 shows three examples of how malicious nodes affect routing success rate and key utilization. (a) Malicious nodes spread incorrect link state information, simultaneously reducing key utilization efficiency and the success rate of key distribution. The yellow and green routes represent the ideal paths from G to F and from A to E, respectively, which would maximize the utilization of all available resources in the current network. However, guided by the false link information introduced by malicious nodes, node I performs incorrect routing, causing the key distribution to fail for the G-to-F and A-to-E paths. (b) Malicious nodes make routing decisions in their own way, misrouting key forwarding along suboptimal paths. Such behavior increases unnecessary key consumption and reduces the demand completion ratio. The yellow route represents the ideal shortest route from A to C. Due to incorrect forwarding by malicious nodes (red route), node B can only reach C via the green route, which consumes more key resources. (c) Malicious nodes forge communication demands and compete for resources with legitimate nodes. For example, in the figure, the legitimate path from node A to G is disrupted by a forged demand from the malicious node along the F-to-E path, resulting in routing failure for A to G. Their attack capabilities follow the attacker’s assumptions below:

In general, the attacker’s strategy space is unbounded: beyond the three representative modes we study, collusion, adaptivity, and mixed strategies induce a combinatorial explosion of behaviors. Although we propose security-oriented mitigations, precisely quantifying the impact of each attack family—e.g., the incremental QKD-key consumption under an adaptive, stateful adversary—remains intrinsically difficult. Realistic attacks are often contingent and history-dependent (reacting to topology/key-rate fluctuations and defensive responses), making exhaustive enumeration or simulation impractical. Nevertheless, a small set of canonical attack modes suffices to reveal the missing fundamental security properties of QKD networks and to characterize the principal impact pathways on reliability and key utilization.

In a practical routing protocol, each node determines the next-hop node based on its locally available routing state. Typically, a single node does not have complete knowledge of all the parameters in the above optimization formulation. While the optimization model aims to minimize key consumption and ensure fairness, malicious nodes can directly distort the optimization variables. The influence of the three attack modes can be summarized as follows:

1. In Attack Mode 1, malicious nodes alter a part of the routing key-state values $S_{e}^{t}$ and $G_{e}^{t}$.
2. In Attack Mode 2, malicious nodes abuse their control to arbitrarily alter the flow variables $f_{e_{in}}^{t}$ and $f_{e_{out}}^{t}$, thereby misrouting key forwarding along suboptimal paths.
3. In Attack Mode 3, malicious nodes can add external demand variables $m_{ij}^{t}$.

Currently, there is no existing research on the design of routing protocols specifically addressing the presence of malicious nodes in QKD networks. This paper explores the impact of malicious nodes on QKD networks routing and proposes a novel mechanism to mitigate these effects.

## 3. Trust-Aware Causal Consistency Routing Against Malicious Nodes

### 3.1. Overview

First, we provide an overview of our proposed scheme and discuss why addressing routing issues in the presence of malicious nodes requires consistency. In QKD networks, routing decisions typically depend on the state of the key pool. Here, we categorize the state of the key pool into three parts: the current storage amount $S_{e}^{t}$ on the link *e*, the generated amount $G_{e}^{t}$ expected in the next time interval and the amount to be consumed $K_{e}^{t}$ in the subsequent time interval.

Under normal circumstances, each honest node determines $K_{e}^{t}$ according to its routing table and the received requests, which may originate either from upstream nodes or from its own demands. When all nodes in the network behave honestly, routing table updates are typically accurate, and the demands communicated from other nodes are trustworthy. However, in the presence of malicious nodes, neither of these conditions can be guaranteed, causing the decision-making process for $K_{e}^{t}$ to become suboptimal or even erroneous.

**Figure 2:**
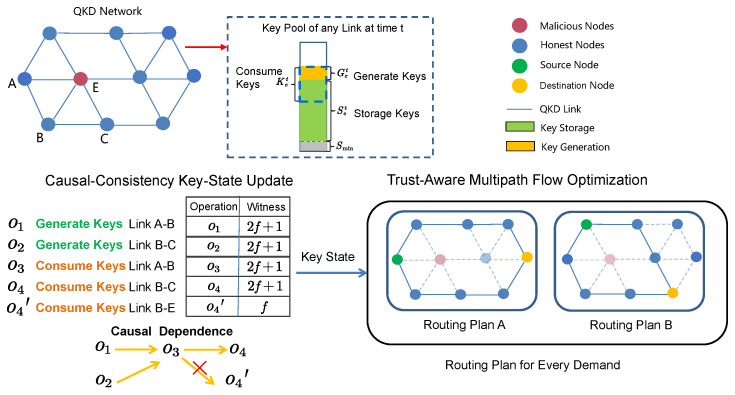
Overview of Trust-Aware Causal Consistency Routing with malicious nodes. Red nodes are malicious, and blue nodes are honest. The symbol × indicates that the operation witness is insufficient and thus untrusted.

We address these challenges from two complementary perspectives, as illustrated in Figure 2. First, to ensure that every honest node can reliably observe network key-state changes despite malicious interference, we design a Causal Consistency Key-State Update mechanism. This mechanism extends each node’s visibility at low cost and embeds a witness protocol that ensures a majority of nodes reach agreement on each link’s key state. Second, we propose a Trust-Aware Multi-path Flow Optimization scheme because some key-state reports may not trustworthiness under adversarial conditions. This approach incorporates the reported trustworthiness of key-state information as an explicit optimization variable and replaces traditional single-path routing with a multi-path strategy, thereby improving robustness against malicious behavior.

### 3.2. Causal Consistency Key-State Update

We propose a secure and causally consistent routing protocol designed for distributed systems with up to *f* malicious nodes. Causal consistency is a consistency model in distributed systems that preserves the order of causally related operations across all nodes, while allowing concurrent operations that are not causally related to be observed in different orders [36].

The role of this scheme is to leverage causal consistency to eliminate inconsistent key states disseminated by malicious nodes, while simultaneously maintaining a consistent view of routing plan updates.

Definition (Causal Consistency). Let $o_{1}$ and $o_{2}$ be two operations in a history *H*. We say $o_{1}$ causally precedes $o_{2}$, denoted $o_{1}\to o_{2}$, if one of the following holds:

1. Program order: They are issued by the same process and $o_{1}$ must appear before $o_{2}$ or $o_{2}$ depending on the result of $o_{1}$.
2. Reads-from: $o_{1}$ writes to some object, and $o_{2}$ later reads that same object value.
3. Transitivity: There is an intermediate operation $o^{\prime }$ such that $o_{1}\to o^{\prime }$ and $o^{\prime }\to o_{2}$.

A system is causally consistent if, whenever $o_{1}\to o_{2}$ in the global history, every correct node observes $o_{1}$ before $o_{2}$.

Unlike strong consistency models that require total ordering of operations, causal consistency only enforces partial ordering among operations that have a causal dependence. This weaker consistency is particularly useful in large-scale or low-latency environments where enforcing total order may be prohibitively expensive [37,38].

Examples in Routing Protocols:

1. Key Consumption Across Multiple Paths: Suppose two operations $o_{1}$ and $o_{2}$ consume keys on the same link’s key pool. If $o_{2}$ depends on the key amount reduced by $o_{1}$, then $o_{1}\to o_{2}$. Causal consistency requires that no correct node applies $o_{2}$ without first having observed the updated capacity after $o_{1}$.
2. Key Regeneration and Route Advertisement: Consider a link (u,v) whose key resources are initially exhausted (capacity =0) and then replenished at time *t* through a new round of key generation. This regeneration is an operation $o_{1}$. Later, a routing protocol advertises (u,v) to be used in a new route $o_{2}$. Because $o_{2}$ depends on the prior knowledge that (u,v) has available key material, we have $o_{1}\to o_{2}$. Causal consistency ensures that every node that accepts $o_{2}$ must have first observed $o_{1}$.

The routing protocol determines the next key consumption $K^{t+1}$ based on key storage $S^{t}$, while $S^{t}$ is in turn influenced by $K^{t-1}$. This establishes a causal relationship between the current routing state and key consumption decisions. By enforcing causal consistency, a routing protocol avoids erroneous decisions, such as allocating non-existent link capacity or using outdated key values. Though weaker than strong sequential consistency, causal consistency typically demands significantly lower coordination communication costs, making it especially well-suited for large-scale routing.

#### 3.2.1. Protocol Definition and Setup

We consider a QKD network of *n* nodes, among which at most *f* are malicious (arbitrary adversarial behavior), and the remaining nodes are correct. We assume n≥3f+1. To prevent interference from malicious nodes in the routing protocol while preserving causal consistency, our approach employs the following mechanisms:

Vector Clock: We maintain a single vector clock (VC) for each node, denoted as $\mathrm{VC}\in N^{\left|V\right|}$. Each entry VC[k] represents the latest version of events (either key-state changes or routing-plan updates) that originated from node *k*. In this unified design, both key generation/consumption events and routing decisions are treated uniformly as operations that advance the VC by one unit.

This unified VC ensures that all key-state changes and routing-plan updates are causally ordered across honest nodes, preventing malicious nodes from propagating inconsistent states or causing honest nodes to apply updates out of order.

Witness Mechanism: We use a threshold-based witness approach to validate updates. Specifically,

- If an update receives fewer than f+1 witnesses, it is considered potentially forged and not acknowledged.
- If it has at least f+1 witnesses, it is partially validated but not yet committed.
- Only when an update accumulates at least n−f witnesses can it be safely delivered and causally applied.

Initial Setup: Each node is initialized with the following:

- A vector clock $\mathrm{VC}\in N^{\left|V\right|}$, with all entries initially set to zero.
- A pending queue for delayed updates with unmet dependencies.
- A witness_log for storing witnessed updates from other nodes.
- A commit_log for recording locally applied and confirmed updates.
- A view of the current round number *r*, with leader ℓ(r)=rmodn.

Each node starts with the initial key states of its directly connected links and no active route plan. As updates propagate, both key-state events and routing-plan updates advance the unified VC consistently across the network.

Message Format: The protocol defines two types of messages: update messages, which propagate state changes and vector clocks, and witness acknowledgments, which confirm the authenticity of updates.

- Update Message:
  M=(type,Update_set,vc,deps,sig)
  An update message is generated whenever a node performs a key-state change or a routing-plan update. It carries the following:
  – The incremented vc field, which reflects the updated vector clock entry corresponding to the event.
  – A dependency set, deps, which is a snapshot of the sender’s current VC. This ensures causal ordering: if a receiver’s local VC is behind any entry in deps, the update is placed into the pending queue until the missing dependencies are applied.
  – The Update_set, which contains either the local key-state increments on adjacent links or a multi-path routing plan involving those links.
  – A type field indicating whether the update is a KeyState or RoutePlan.
  – A sig field with the sender’s digital signature, ensuring message integrity and authenticity. Signature implementation can follow the schemes described in [39] or [40].
- Witness Acknowledgment:
  W=(msg_hash,sig,sender_id)
  A witness acknowledgment confirms receipt and validation of an update. It includes the hash of the acknowledged update, the witness node’s signature, and the sender’s identifier.

#### 3.2.2. Causal Consistency Key-State Update in Round *r*

Leader Behavior of Round *r*: The leader ℓ(r) coordinates route planning and update validation by performing the following:

1. Check Key-State Witnesses
  - For each link, verify that the latest KeyState updates satisfy all dependencies in deps.
  - Filter the key states that have accumulated more than f+1 witnesses. Since key states are broadcast by nodes, we check whether both adjacent nodes *i* and *j* have witness counts exceeding f+1. If this condition is satisfied, the key state of link (i,j) is included in the valid key states.
2. Compute and Broadcast New Plan
  - Computes a new multi-path route plan as the method in Section 3.3 based on the valid key states and communication demands.
  - Records all edges scheduled to consume keys, along with the corresponding key consumption amounts in the update_set. Records in deps the vector-clock values of these edges from their most recent routing states. Forms a batched RoutePlan update message:
    M=(type=RoutePlan,update_set,vc,deps,sig)
  - Broadcasts the new RoutePlan message to all nodes for witness collection.

Regular Node Behavior in Round *r*. Each non-leader node performs the following actions upon receiving requests or updates from the leader:

1. Update Local State:
  - Broadcasts the current local state and communication demands, including the following:
    – Latest key states on adjacent links.
    – Communication demands with the current node as the source.
2. Receive and Verify Route Plan:
  - Upon receiving a RoutePlan update from the leader, verify the following:
    – The leader’s signature.
    – Whether the update’s deps are satisfied by local vector clocks.
    – Whether the leader issued conflicting route plans (equivocation).
  - If verification passes, sends a Witness Acknowledgment:
    W=(plan_hash,sig,sender_id)
  - If conflict is detected, broadcasts a Conflict Witness Acknowledgment:
    W=(plan_hash_1,plan_hash_2,sig,sender_id)
3. Apply Finalized Updates:
  Once any KeyState or RoutePlan update receives at least n−f witness acknowledgments and all dependencies are met, the node performs the following:
  - Verifies its causal dependencies. Specifically, for each entry deps[k] in the update *U*, the node checks whether:
    VC[k]≥deps[k]
  - If any dependency is not yet satisfied, the update is placed into a pending queue, else move the update to commit_log.
  - Applies the corresponding key state change to local state.

### 3.3. Trust-Aware Multi-Path Flow Optimization

In this section, we present the design of an optimization method tailored for scenarios involving malicious nodes. This method builds upon the approach introduced in Section 3.2, with the following two key enhancements:

1. Using link state perception discrepancies as a measure of trustworthiness, allowing the system to quantify the reliability of each link based on the consistency of reported states from connected nodes.
2. Extending the original routing strategy to a multi-path routing formulation, which enhances resilience against malicious behavior by diversifying key distribution paths and reducing reliance on any single potentially compromised route.

Sets and notation. Let *V* be the node set and *E* the directed edge set (undirected links are modeled as two opposite arcs). For v∈V, denote $\delta^{+}\left(v\right)=\left\{e=\left(v,\cdot \right)\in E\right\}$ and $\delta^{-}\left(v\right)=\left\{e=\left(\cdot ,v\right)\in E\right\}$.

Parameters. $m_{ij}^{t}\;\geq \;0$: offered (maximum) demand from *i* to *j* at time *t*; *f*: upper bound on malicious nodes; $S_{\min}$: minimum reserved storage; $C_{e}^{t}\;\in \left[0,1\right]$: trust score of edge *e* at time *t*; $G_{e}^{t}\;\geq \;0$: key generation on *e* at time *t*; $S_{e}^{t}\;\geq \;0$: Key pool capacity or edge capacity available *e* at time *t* λ≥0: fairness weight. μ≥0: demand weight. β≥0: trust-aware weight.

Decision variables. ${\hat{m}}_{ij}^{t}\;\geq \;0$: served/completed demand from *i* to *j* at time *t*; $F_{e,ij}^{t}\;\geq \;0$: flow of commodity (i,j) on edge *e* at time *t*; $\Delta^{t}\;\geq \;0$: maximum per-source deviation at time *t*; $y_{a,ij}^{t}\;\in \;\left\{0,1\right\}$: reliability flow on arc *a* (in the node-splitting graph) for demand (i,j) at time *t*.

Each node reports its own key state, and there will be reports from two adjacent nodes. We denote these as $\left(G_{e_{A}}^{t},S_{e_{A}}^{t}\right)$ and $\left(G_{e_{B}}^{t},S_{e_{B}}^{t}\right)$. In theory, these two sets of variables should be consistent. However, if one of the nodes is a malicious node and has falsified its key state, the discrepancy between the two sets will increase. Therefore, we can use the error between these two sets of variables as a reference parameter to determine the trustworthiness of the link. The calculation is as follows:

(8) $$\begin{array}{c} C_{e}^{t}=\frac{\left(\min\left(W_{e_{A}}^{t},W_{e_{B}}^{t}\right)-f\right)}{n-f}\cdot exp\left(-\beta {\left(G_{e_{A}}^{t}+S_{e_{A}}^{t}-G_{e_{B}}^{t}-S_{e_{B}}^{t}\right)}^{2}\right) \end{array}$$

where $C_{e}^{t}$ is a trusted level of edge *e* at time *t*. $\min(W_{e_{A}}^{t},W_{e_{B}}^{t})$: Witness of the key state of node A and B on edge *e* at time *t*. $S_{e}^{t}$: Key pool capacity or edge capacity available *e* at time *t*. $G_{e}^{t}$: Key generation rate on edge *e* at time *t*.

Objective.

(9) $$\min\;\sum_{t}\sum_{i,j\in V}F_{e,ij}^{t}\;+\;\lambda \sum_{t}\Delta^{t}\;-\;\mu \sum_{t}\sum_{i,j\in V}{\hat{m}}_{ij}^{t}$$

1. Multiple disjoint path constraints. Construct $G^{\prime }=\left(V^{\prime },E^{\prime }\right)$ by splitting each v∈V into $v^{\mathrm{in}},v^{\mathrm{out}}$ with a node-arc $\left(v^{\mathrm{in}},v^{\mathrm{out}}\right)$, and for each e=(u,v)∈E add a link-arc $\left(u^{\mathrm{out}},v^{\mathrm{in}}\right)$. Let $\psi \left(e\right)=\left(u^{\mathrm{out}},v^{\mathrm{in}}\right)$ map *e* to its link-arc.

(10) $$\sum_{a\in \delta^{\prime -}\left(w\right)}y_{a,ij}^{t}\;-\;\sum_{a\in \delta^{\prime +}\left(w\right)}y_{a,ij}^{t}\;=\;\left\{\begin{array}{cc} \;\;p_{ij}^{t}, & w=j^{\mathrm{in}},\\ -p_{ij}^{t}, & w=i^{\mathrm{out}},\\ \;\;0, & \mathrm{otherwise}, \end{array}\right.\;\forall w\in V^{\prime },\;\forall i,j\in V,\;\forall t.$$

(11) $$\sum_{a:\;a=(v^{\mathrm{in}},v^{\mathrm{out}})}y_{a,ij}^{t}\;\leq \;1,\;\forall v\in V\setminus \left\{i,j\right\},\;\forall i,j\in V,\;\forall t.$$

(12) $$p_{ij}^{t}\in Z_{+},\;y_{a,ij}^{t}\in \left\{0,1\right\},\;p_{ij}^{t}\geq f+1,\;\forall a\in E^{\prime },\;\forall i,j,\;\forall t.$$

2. Flow Conservation constraints.

(13) $$0\leq {\hat{m}}_{ij}^{t}\leq m_{ij}^{t},\;\forall i,j\in V,\;\forall t.$$

(14) $$F_{ij}^{t}\;=\;\;\frac{{\hat{m}}_{ij}^{t}}{p_{ij}^{t}-f},\;\forall i,j\in V,\;\forall t.$$

3. Trust-aware constraints.

(15) $$0\leq F_{ij}^{t}\;\leq \;\left(S_{\left(u,v\right)}^{t}-S_{\min}\right)\;C_{\left(u,v\right)}^{t}\cdot y_{\psi (u,v),ij}^{t},\;\forall \left(u,v\right)\in E,\;\forall i,j\in V,\;\forall t.$$

4. Key Pool Dynamic constraints.

(16) $$S_{e}^{t+1}\;=\;S_{e}^{t}+G_{e}^{t}-\sum_{i,j\in V}F_{ij}^{t},\;\forall e\in E,\;\forall t.$$

5. Fairness constraints.

(17) $$-\Delta^{t}\;\leq \;\sum_{j\in V}{\hat{m}}_{ij}^{t}-\sum_{j\in V}{\hat{m}}_{kj}^{t}\;\leq \;\Delta^{t},\;\forall i,k\in V,\;\forall t.$$

Allowing a variable number of node-disjoint paths enables the protocol to exploit more independent routes for key distribution, sharply reducing the risk from any single path. Since the number of adversaries is at most *f*, the design can discard up to *f* compromised paths and still meet the target demand, thereby mitigating losses under Attack Mode 1 and Attack Mode 2. In parallel, a fairness constraint balances the served volume across sources, curbing resource hoarding by malicious nodes Attack Mode 3 and preserving efficient key utilization and high demand-completion even in adversarial conditions.

In large, dynamically evolving QKD networks, directly solving the foregoing model as an integer linear program (ILP) yields a prohibitively large number of binary/integer variables and strong cross-commodity couplings, leading to substantial computational burden. We can adopt a preprocessing + dimensionality reduction strategy that compresses edge-level combinatorics to path-level decisions, which significantly accelerates optimization while preserving the essential trust and capacity semantics.

1. We can pre-compute per-link trust levels and, for each demand (i,j,t), enumerate a small set of candidate node-disjoint paths (e.g., *K* trusted shortest/vertex-disjoint paths). In the optimization, binary decisions are attached to paths (select/not-select) rather than to edges.
2. We can apply a trust threshold to retain only high-confidence candidate paths before optimization, thereby removing low-trust routes that would be down-weighted or eliminated by the solver anyway. In addition, the number of required node-disjoint paths can be fixed (e.g., $p_{ij}^{t}\equiv p_{0}$), avoiding a combinatorial search. This restriction further shrinks the integer feasible set and stabilizes solve times under rapid trust/key-rate fluctuations.
3. We can relax the global fairness coupling by choosing a larger fairness variable $\Delta^{t}$ (or by softening fairness into a penalty in the objective with an increased weight λ). This allows a controlled relaxation of fairness to substantially reduce solve time and improve stability.

## 4. Simulation

### 4.1. Simulation Setup

QKD Network Setup: To obtain statistical simulation results under varying network topologies, we used a random network topology and performed multiple experiments with a varying number of nodes ranging from 10 to 50. The number of malicious nodes ranges from 10% to 30% of the total nodes, and these nodes are selected randomly. The generation rate of QKD keys and the configuration of the key pool are referenced in [41]. The QKD network is randomly generated as follows: given the total number of nodes and the proportion of malicious nodes, edges are iteratively added between randomly selected node pairs with a probability of 0.2, until the network’s vertex connectivity exceeds the number of malicious nodes. The simulation in different setups will be run 10 times with different random seeds, and the average values of these trials represent the simulation results [16]. This can be used to more accurately verify the effectiveness of the proposed scheme in this paper under different topologies.

Key Requirement Setup: We established a fixed packet size of 1 kb. A Poisson distribution was employed to model the frequency of packet transmission between any given pair of nodes [42]. Consequently, the times of key distribution frequency of a node pair were modeled as a Poisson distribution $d\left(\lambda \right)$ with a mean value of λ=0.6/s. Total simulation time t=50 s.

Routing Protocol Setup: Three protocols are used in the simulation. The first is the standard OSPF protocol, serving as a baseline. The second is a multi-path variant of OSPF, which replaces traditional path selection with an optimization-based multi-path routing algorithm, while retaining OSPF’s original routing state awareness. The third is our proposed protocol. The second protocol adopts the same optimization algorithm for path planning as our proposed protocol, but it lacks causal consistency in key state synchronization and thus cannot employ the trust-aware mechanism. The comparison between Protocol 1 and Protocol 2 highlights the benefits of multi-path routing optimization, while the comparison between Protocol 2 and Protocol 3 reveals the advantages introduced by causal consistency in routing state awareness and the trust-aware mechanism.

Attack Modes Setup:

1. Inconsistent routing broadcasts (Attack Mode 1): Malicious nodes disseminate conflicting routing information during state broadcasts. On each broadcast, with probability 50%, the attacker perturbs the routing state by a random percentage (uniform from [−0.30, +0.30]).
2. Incorrect forwarding paths (Attack Mode 2): Malicious nodes are free to make routing decisions in their own way, without being constrained by the protocol. The malicious nodes randomly select a suboptimal feasible next hop.
3. Excessive key demands (Attack Mode 3): Malicious nodes generate inflated key requests to exhaust available resources. Every pair of non-adjacent malicious nodes generates additional demands (same λ to honest nodes).

### 4.2. Simulation Results

Our simulation results focus on two key metrics, the demand completion ratio and the key utilization efficiency, as they jointly reflect the protocol’s ability to maintain robustness and resource efficiency under malicious attacks.

#### 4.2.1. Simulation Results About Demand Completion Ratio

**Figure 3:**
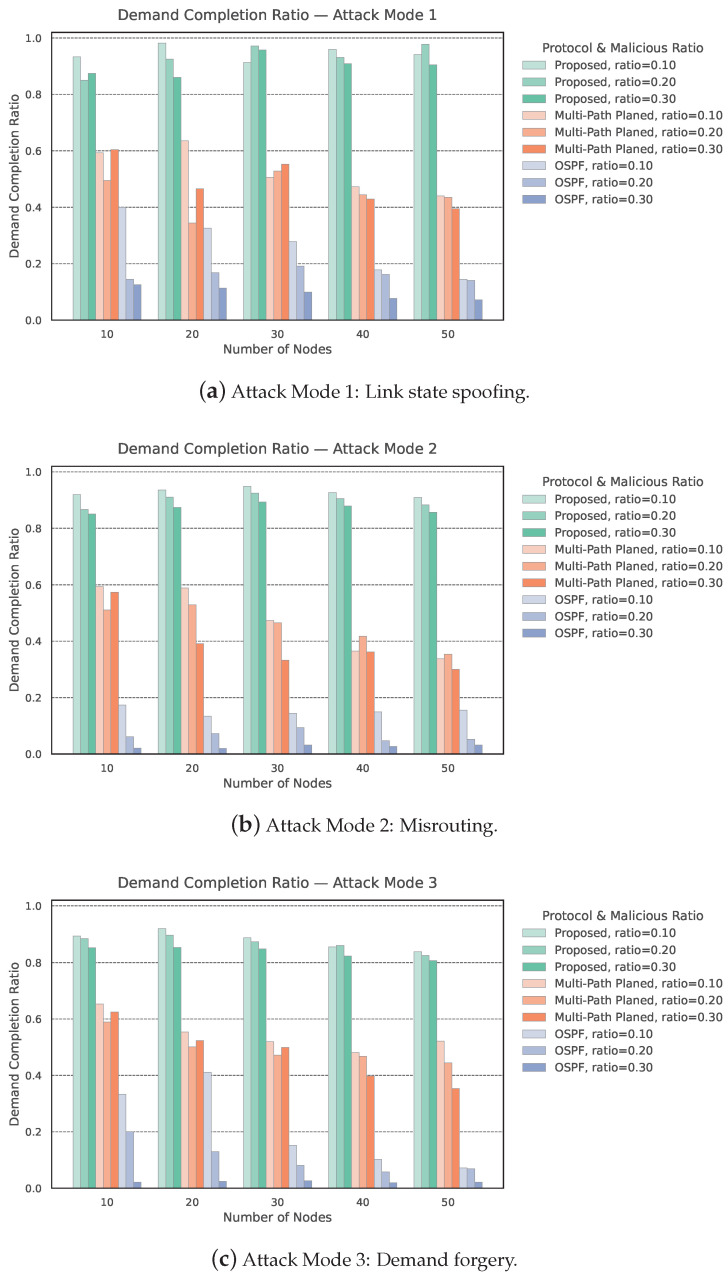
The simulation results of the demand completion ratio for three different protocols, with the number of nodes ranging from 10 to 50 and malicious node ratios of 0.1, 0.2, and 0.3. (**a**–**c**) show the demand completion ratios under the three attack modes.

As shown in Figure 3, we demonstrated the demand completion ratio (DCR) for three protocols—Proposed, Multi-Path Planned, and OSPF—under varying network size *N*, malicious nodes ratio ϕ, and attack mode *m*.

Impact of Malicious Node Ratio on DCR. We first analyze the impact of the malicious node ratio ϕ on DCR. As illustrated in Figure 3a, under Attack Mode 1 and a low adversarial presence (ϕ=0.1), the Proposed protocol sustains DCR above 0.91 even as the network size grows from N=10 to N=50. By contrast, Multi-Path Planned achieves only [0.65,0.44], while OSPF degrades sharply to below 0.14 at N=50. When ϕ increases to 0.3 (still m=1), the Proposed scheme’s DCR declines modestly (≥0.86), whereas OSPF drops to 0.07–0.13 under the same setting.

Effect of Network Size on DCR. Second, we investigate the effect of the network size *N* on DCR. Under Attack Mode 2, increasing *N* amplifies both overall key consumption and the negative impact of larger ϕ. For ϕ=0.1, the Proposed protocol consistently maintains DCR >0.90 across N={10,20,30,40,50}, while for ϕ=0.3, its DCR decreases only to average from 0.86, demonstrating resilience to ratio of malicious nodes. In contrast, Multi-Path Planned falls to 0.30 when ϕ = 0.3 at N=50, and OSPF collapses below 0.02 in the same conditions. These results show that a larger network size influences all protocols, but the Proposed scheme mitigates this scaling effect more effectively.

Consistency Trend Across Three Attack Modes. Finally, Figure 3c further confirms the robustness of the Proposed protocol in Attack Mode 3. For instance, at ϕ=0.2 and N=30, the Proposed method achieves DCR 0.92 with moderate key usage, while OSPF drops below 0.09. This consistency across all three attack modes (m∈{1,2,3}) highlights that Causal-Consistent Key-State Updates substantially enhance DCR resilience against malicious node behaviors.

#### 4.2.2. Simulation Results About Key Utilization Efficiency

**Figure 4:**
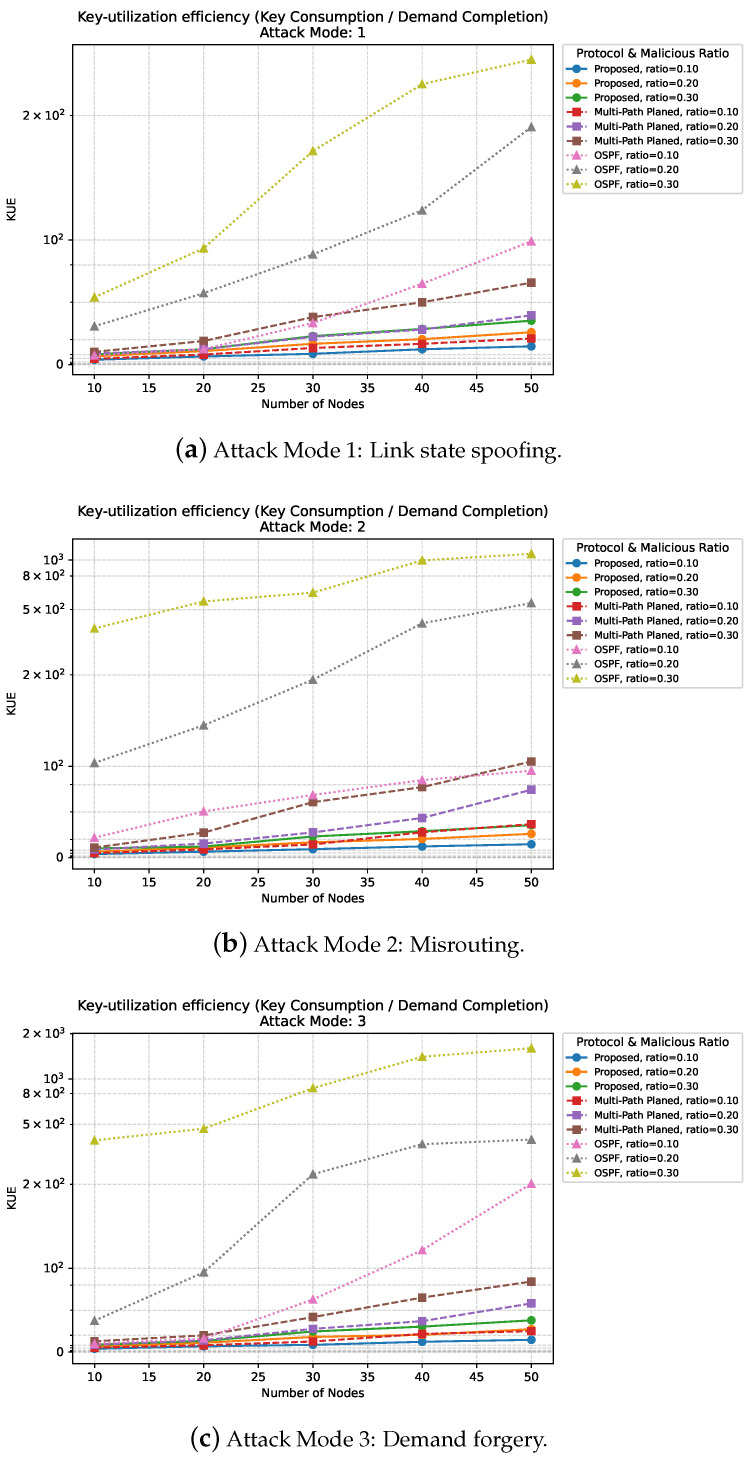
The simulation results of the key utilization efficiency for three different protocols, with the number of nodes ranging from 10 to 50 and malicious node ratios of 0.1, 0.2, and 0.3. (**a**–**c**) show the key utilization efficiency under the three attack modes.

As shown in Figure 4, we further examine the relationship between and the number of nodes under different malicious node ratios. Note that a smaller KUE value indicates higher efficiency, since fewer keys are consumed per successfully completed demand.

Impact of Malicious Node Ratio on KUE. We first analyze the impact of the malicious node ratio ϕ. From Figure 4a, the KUE η of the proposed protocol remains consistently low compared to baselines, even as the adversarial intensity increases. For ϕ=0.1, Proposed η increases from ≈3.86 at N=10 to ≈14.62 at N=50; in contrast, Multi-Path Planned rises from ≈5.93 to ≈28.90, and OSPF escalates sharply from ≈7.34 to ≈98.94. At ϕ=0.3, Proposed η increases from ≈9.27 (N=10) to ≈35.24 (N=50), whereas Multi-Path Planned and OSPF reach ≈65.74 (N=50) and ≈355.24 (N=50), respectively.

Effect of Network Size on KUE. Second, we study the effect of network size *N*. Under forwarding-deviation attacks (m=2), larger networks amplify the KUE penalty imposed by malicious nodes. For example, in Figure 4b for ϕ=0.1, Proposed η grows moderately from ≈3.91 at N=10 to ≈14.65 at N=50, while Multi-Path Planned rises from ≈5.92 to ≈36.65 and OSPF from ≈21.85 to ≈95.18. At ϕ=0.3, Proposed remains below ≈37.55 even at N=50, whereas Multi-Path Planned and OSPF surge to ≈105.29 and ≈1090.47, respectively. This indicates that, although KUE deteriorates for all protocols as both *N* and ϕ increase, the Proposed scheme mitigates the combined scaling and adversarial effects far more effectively.

KUE Trends Under Three Malicious Attack Modes. Finally, Figure 4c confirms similar trends under demand-forging attacks (m=3). For ϕ=0.1, Proposed’s η grows from ≈4.03 at N=10 to ≈16.67 at N=50, while Multi-Path Planned and OSPF jump to ≈25.04 and ≈202.13, respectively. At ϕ=0.3, Proposed maintains η≈8.79 (N=10) and ≈35.88 (N=50), whereas the baselines exceed ≈84.28 (Multi-Path N=50) and ≈1601.22 (OSPF N=50). Hence, across all three attack modes, Causal Consistency Key-State Updates not only stabilize DCR but also keep KUE growth within moderate bounds, demonstrating robust resistance to adversarial behaviors.

## 5. Conclusions

In this work, we introduced a novel Trust-Aware Causal Consistency Routing framework for QKD networks that integrates Causal Consistency Key-State Updates and Trust-Aware Multi-path Flow Optimization. The framework is specifically designed to sustain reliable performance even when part of the network is compromised by malicious relay nodes.

Our evaluation on networks of up to 50 nodes with malicious fractions ϕ∈{0.1,0.2,0.3} under three attack modes highlights the clear advantage of our scheme. The proposed protocol consistently sustains a high demand completion ratio (DCR), averaging 0.90 (range 0.81–0.98), while keeping key utilization efficiency (KUE) low at an average of 16.6 keys per completed demand. By contrast, Multi-Path Planned achieves only moderate DCR (mean 0.48) and consumes nearly twice as many keys (KUE mean 30.8), whereas OSPF collapses to DCR ≤0.12 and wastes hundreds to thousands of keys per demand (KUE mean 296, max 1601). These results clearly demonstrate that our design achieves a near-optimal balance: high reliability (DCR) with efficient resource usage (KUE), even under adversarial conditions.

This balance between DCR and KUE is crucial in QKD networks, since completing more demands at the cost of excessive key consumption is unsustainable, while conserving keys without meeting demands undermines service availability. Our framework demonstrates that it is possible to simultaneously preserve both, providing robust, efficient routing against a wide range of malicious behaviors.

Nevertheless, this study should be seen as an initial step in routing with malicious nodes. Real adversaries may employ unbounded strategies, including collusion, adaptivity, and mixed strategies that yield a combinatorial explosion of behaviors. One possible mitigation strategy is using AI-driven/reinforcement learning/planning agents to synthesize adaptive, colluding attack policies against our defenses, yielding more realistic dynamic strategy games. In the future, we will extend beyond the three attack modes studied here, incorporating broader adversarial models, adaptive countermeasures, AI-driven adversarial dynamic strategy and game-theoretic adversarial approaches to further strengthen the resilience of QKD routing in hostile environments.

## Data Availability

The data presented in this study are available on request from the corresponding author.

## Abbreviations

The following abbreviations are used in this manuscript:

QKD	Quantum Key Distribution
DCR	Demand Completion Ratio
KUE	Key Utilization Efficiency
ITS	Information-Theoretic Security
TLS	Transport Layer Security
IEEE	Institute of Electrical and Electronics Engineers
ACM	Association for Computing Machinery
PRX	Physical Review X
SIAM	Society for Industrial and Applied Mathematics
RFC	Request for Comments
OSPF	Open Shortest Path First
IS-IS	Intermediate System to Intermediate System
AI	Artificial Intelligence
ILP	Integer Linear Program
